# Cross-Cultural Identification of Acoustic Voice Features for Depression: A Cross-Sectional Study of Vietnamese and Japanese Datasets

**DOI:** 10.3390/bioengineering13010033

**Published:** 2025-12-27

**Authors:** Phuc Truong Vinh Le, Mitsuteru Nakamura, Masakazu Higuchi, Lanh Thi My Vuu, Nhu Huynh, Shinichi Tokuno

**Affiliations:** 1 Faculty of Public Health, University of Medicine and Pharmacy at Ho Chi Minh City, Ho Chi Minh City 749000, Vietnam; vtmlanh@ump.edu.vn; 2 Graduate School of Health Innovation, Kanagawa University of Human Services, Yokosuka 241-0815, Japan; m.nakamura-kgr@kuhs.ac.jp (M.N.); higuchi-2mf@kuhs.ac.jp (M.H.); s.tokuno-wm2@kuhs.ac.jp (S.T.); 3 School of Finance, University of Economics Ho Chi Minh City, Ho Chi Minh City 722700, Vietnam; 22004025@student.westernsydney.edu.vn

**Keywords:** depression, voice biomarkers, acoustic features, cross-cultural analysis, machine learning

## Abstract

Acoustic voice analysis demonstrates potential as a non-invasive biomarker for depression, yet its generalizability across languages remains underexplored. This cross-sectional study aimed to identify a set of cross-culturally consistent acoustic features for depression screening using distinct Vietnamese and Japanese voice datasets. We analyzed anonymized recordings from 251 participants, comprising 123 Vietnamese individuals assessed via the self-report Beck Depression Inventory (BDI) and 128 Japanese individuals assessed via the clinician-rated Hamilton Depression Rating Scale (HAM-D). From 6373 features extracted with openSMILE, a multi-stage selection pipeline identified 12 cross-cultural features, primarily from the auditory spectrum (AudSpec), Mel-Frequency Cepstral Coefficients (MFCCs), and logarithmic Harmonics-to-Noise Ratio (logHNR) domains. The cross-cultural model achieved a combined Area Under the Curve (AUC) of 0.934, with performance disparities observed between the Japanese (AUC = 0.993) and Vietnamese (AUC = 0.913) cohorts. This disparity may be attributed to dataset heterogeneity, including mismatched diagnostic tools and differing sample compositions (clinical vs. mixed community). Furthermore, the limited number of high-risk cases (n = 33) warrants cautious interpretation regarding the reliability of reported AUC values for severe depression classification. These findings suggest the presence of a core acoustic signature related to physiological psychomotor changes that may transcend linguistic boundaries. This study advances the exploration of global vocal biomarkers but underscores the need for prospective, standardized multilingual trials to overcome the limitations of secondary data analysis.

## 1. Introduction

Depression constitutes a primary driver of global disability, affecting approximately 280 million individuals and contributing to over 13% of all disability-adjusted life-years [1,2]. Despite the availability of evidence-based interventions, a significant treatment gap exists, particularly in low- and middle-income countries (LMICs). In these regions, more than 75% of affected individuals remain untreated due to a systemic shortage of mental health professionals and the pervasive social stigma associated with psychiatric conditions. In Vietnam, cultural barriers and resource constraints often impede large-scale screening efforts, leading to late-stage diagnosis and exacerbating both individual suffering and the broader economic burden [3].

Conventional diagnostic frameworks, including the Hamilton Rating Scale for Depression (HAM-D) and the Patient Health Questionnaire-9 items, remain the clinical gold standard; however, they are inherently limited by subjective interpretation and recall bias [4]. Furthermore, the administration of these instruments in primary-care settings is often hindered by the requirement for specialized training and significant time investment. While objective alternatives such as biochemical markers (e.g., salivary cortisol, blood gene-expression assays) and neuroimaging offer higher precision, their invasive nature and high operational costs render them impractical for widespread deployment in resource-limited settings [5]. This disparity underscores the urgent necessity for non-invasive, cost-effective, and objective screening modalities that can be seamlessly integrated into routine clinical workflows.

The pursuit of objective diagnostic tools has evolved from biochemical assays to sophisticated neurological assessments. Recent innovations, such as the NeuroFeat framework, have demonstrated that adaptive feature engineering—specifically utilizing the Logarithmic-Spatial Bound Whale Optimization Algorithm (L-SBWOA)—can refine feature spaces to achieve classification accuracies reported as high as 99.22% [6]. While deep learning models often demonstrate superior performance in such contexts, traditional machine learning approaches remain highly relevant due to their lower computational requirements and superior interpretability within clinical environments [7]. While these neuroimaging-based frameworks show high accuracy, the need for specialized hardware limits their scalability. Consequently, acoustic voice analysis has emerged as a compelling alternative, reconciling the tension between high-precision computational modeling and the need for accessible, non-invasive screening tools.

The field of vocal biomarkers is advancing rapidly, with contemporary research prioritizing the development of robust, multilingual models suitable for both clinical and remote monitoring [8,9]. A vocal biomarker is defined as an objective feature, or combination thereof, derived from an audio signal that correlates with clinical outcomes, thereby facilitating risk prediction and symptom monitoring [8]. Speech production involves complex neuromotor coordination that reflects subtle affective shifts; core depressive symptoms, such as psychomotor retardation and anhedonia, manifest through altered acoustic properties, including reduced fundamental frequency (F0) variability, prolonged pauses, diminished intensity, and spectral shifts [10,11,12,13]. Crucially, these vocal characteristics are largely involuntary, mitigating the risk of deliberate response bias inherent in self-report measures.

Acoustic investigations typically categorize potential biomarkers into prosodic features, perturbation measures, and spectral qualities. Clinical observations consistently characterize the speech of depressed individuals as slow, monotonous, and breathy [14,15]. Quantitative studies have identified reduced mean F0, increased pause duration, and lower speech rates as significant indicators [8]. Furthermore, perturbation metrics such as Jitter (frequency variation), Shimmer (amplitude variation), and the Noise-to-Harmonics Ratio are frequently utilized, with sustained vowel phonation studies suggesting that amplitude variability is highly discriminant in identifying depressive states [16]. Spectral features, particularly Mel-Frequency Cepstral Coefficients (MFCCs), have also shown promise. For instance, the second dimension (MFCC 2) has been identified as an efficient classifier, reflecting physiological changes in the vocal tract and laryngeal motor control [11].

Despite these technological strides, the inherent variability of human speech—compounded by linguistic and cultural nuances—poses a significant challenge to the cross-linguistic generalizability of automated systems [9,17]. The phonetic structure of a language is a central confounder; for example, tonal languages like Vietnamese utilize pitch lexically, which differs fundamentally from the prosodic structures of non-tonal languages like English or Japanese [12,18]. Research has indicated that the predictive reliability of MFCCs varies across Chinese and Japanese cohorts, likely due to language-specific articulation patterns [11,19]. Moreover, while some markers like articulation rate show cross-linguistic consistency, others—such as pausing behavior—may be significant only in specific linguistic groups [20].

Recent methodological efforts have sought to identify translinguistic vocal markers. Large-scale longitudinal initiatives, such as the RADAR-MDD project spanning English, Dutch, and Spanish, have consistently linked depression severity to decreased speech rate and intensity, suggesting psychomotor impairment as a universal feature [20]. Advanced frameworks, including the multi-lingual Minimum Redundancy Maximum Relevance (ml-MRMR) algorithm and lightweight multimodal fusion networks, have been developed to enhance model generalization across diverse languages such as Turkish, German, and Korean [21,22]. Furthermore, investigations into non-verbal semantic patterns suggest shared experiential states of depression across different cultures [23].

A critical yet frequently underemphasized challenge is dataset heterogeneity and the resultant biases stemming from varied recruitment contexts. Systematic reviews highlight that environmental factors (e.g., recording quality), demographic variances (e.g., age, medication), and methodological inconsistencies (e.g., varying speech tasks) significantly influence model outcomes [8,24]. Specifically, comparing data from university volunteers with that of clinically diagnosed patients can introduce confounding variables related to symptom severity and motivational states [18]. To address these issues, recent studies have employed rigorous statistical designs, such as two-stage meta-analyses and Linear Mixed Effects Models, to account for site-specific variability and within-participant clustering [14,20].

While robust evidence supports the existence of stable vocal phenomena across diverse populations [14], most research remains focused on monolingual, culturally homogeneous cohorts. The generalizability of these biomarkers across the distinct linguistic landscapes of Southeast and East Asia remains largely unexplored [17,25,26]. In Vietnam, although preliminary feasibility studies suggest that voice analysis can distinguish depressed individuals [3,27], a systematic identification of the most discriminative acoustic parameters within the Vietnamese language is currently lacking. Furthermore, direct cross-cultural comparisons—particularly between tonal languages like Vietnamese and non-tonal/pitch-accent languages like Japanese—are absent from the literature, despite evidence that language-specific norms meaningfully influence biomarker performance [17].

To address these empirical gaps, this study aims to: (1) systematically identify a robust set of Vietnamese-specific acoustic features for depression assessment; (2) establish a parallel set of Japanese-specific features; and (3) derive and validate a core set of cross-culturally consistent acoustic features using datasets from both nations. We hypothesize that a subset of acoustic features, primarily those reflecting physiological vocal tract dynamics and neuromuscular control rather than prosodic linguistic elements, will demonstrate consistent discriminative power across both Vietnamese and Japanese contexts. This research seeks to contribute to the development of culturally attuned yet universally applicable vocal biomarkers for mental health.

The remainder of this paper is organized as follows: Section 2 (Materials and Methods) details the datasets, feature extraction pipelines, and validation frameworks; Section 3 (Results) presents the selected feature sets and model performance metrics; Section 4 (Discussion) interprets these findings within the context of cross-linguistic research; and Section 5 (Conclusion) summarizes the clinical and technological implications.

## 2. Materials and Methods

### 2.1. Study Design and Participants

**Figure 1:**
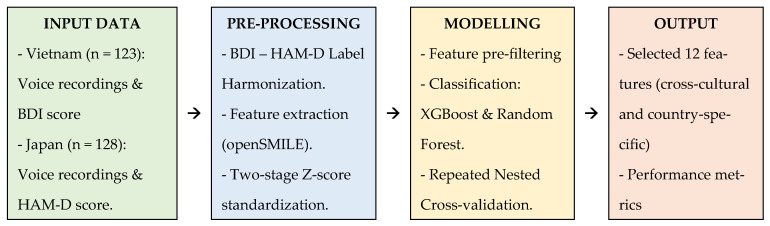
Schematic diagram of the data processing and modeling pipeline.

This study constitutes a secondary analysis, conducted between August 2024 and November 2025, of anonymized voice and clinical datasets previously collected in Vietnam (2022) and Japan (2021). The overall experimental workflow, from data acquisition and pre-processing to model validation, is summarized in Figure 1. The research employs a cross-sectional design focused on identifying robust acoustic biomarkers for depression across two distinct linguistic and cultural contexts.

A primary methodological challenge in this study is the use of disparate diagnostic instruments: a self-report questionnaire (Beck Depression Inventory, BDI) for the Vietnamese cohort and a clinician-administered scale (Hamilton Depression Rating Scale, HAM-D) for the Japanese cohort. This variance introduces potential “label noise” and limits direct score comparability. To mitigate these effects, we implemented a three-tier strategy:

1. Label Harmonization: We established two classification cut-offs (Table 1) designed to approximate comparable clinical risk categories (e.g., ‘At Risk’ vs. ‘High Risk’) rather than relying on raw score equivalence. To ensure robustness, a sensitivity analysis was conducted using alternative thresholds. Nested cross-validation was employed to evaluate the stability of model performance (AUC, sensitivity, specificity) and the consistency of acoustic signatures across these varying definitions.

2. Robust Feature Selection: Our primary hypothesis focused on identifying cross-cultural features that remain discriminative despite methodological variance, treating the different instruments as a realistic confounder in model generalization.
3. Sensitivity Analysis: We conducted analyses on strictly defined high-severity subgroups to ensure the stability of the findings.

Vietnamese Cohort: This dataset originated from a cross-sectional study in Ho Chi Minh City (2022) [27]. A total of 123 participants were recruited via convenience sampling, comprising 100 students and staff from the University of Medicine and Pharmacy and 23 outpatients diagnosed with Major Depressive Disorder (MDD) from Le Van Thinh Hospital. Inclusion criteria required Vietnamese nationality, native language proficiency, and age ≥ 18 years. Depressive symptoms were assessed using the self-reported BDI, referencing the preceding two weeks.

Japanese Cohort: This dataset was obtained from Higuchi et al. (2021) [28], comprising 128 participants recruited from the National Defense Medical College Hospital and Tokyo Medical University Hospital. The cohort included 93 clinically diagnosed MDD patients and 35 healthy controls. MDD patients were recruited during treatment initiation, while the control group consisted of self-reported healthy volunteers (colleagues and staff). Symptom severity for the participants was evaluated using the clinician-administered HAM-D, covering the preceding week.

Ethical approvals were obtained from the respective institutional review boards, and all participants provided written informed consent. The final pooled dataset for this secondary analysis included 251 participants.

### 2.2. Sample Size and Power Consideration

A post hoc power analysis for the primary outcome (Area Under the Curve, AUC) was conducted. With a total sample size of 251 and an alpha level of 0.05, the study was sufficiently powered to detect an effect size corresponding to an AUC of 0.80 (Table S1, Supplementary Materials). While the limited number of high-risk cases (n = 33 across both cut-offs) may reduce the power for severity-specific subgroup analyses, this was addressed through a rigorous nested cross-validation framework.

### 2.3. Voice Recording and Data Acquisition

Voice samples were captured in controlled environments using high-fidelity recording protocols. Participants read aloud a standardized set of predefined phrases. The Vietnamese protocol utilized 16 unique phrases, while the Japanese protocol utilized 21 phrases (Table S2).

Recordings were performed using lavalier microphones (Olympus ME52W, Olympus, Tokyo, Japan) and portable digital recorders (TASCAM DR-100MK3, TEAC Corporation, Tokyo, Japan; Roland R-26 for Roland Corporation, Hamamatsu, Japan). Both devices were pre-validated to ensure no meaningful differences in recording fidelity. All audio data were captured at 24-bit resolution with a 96 kHz sampling rate to ensure high-quality acoustic preservation.

### 2.4. Data Pre-Processing and Acoustic Feature Extraction

Statistical analysis and modeling were executed in R (v4.3.3). Raw audio recordings were manually segmented into individual utterances, resulting in 2091 Vietnamese and 4910 Japanese utterances (Table 2).

A total of 6373 acoustic features were extracted using the openSMILE toolkit (v2.1.0) with the IS13_ComParE configuration. To mitigate inter-speaker and inter-corpus variability, a two-stage z-score standardization procedure was applied:

1. Speaker-level standardization: Features were standardized relative to the individual participant’s mean and standard deviation across all their utterances to control for baseline vocal characteristics.
2. Corpus-level standardization: Features were then standardized using the global mean and standard deviation of the full dataset to ensure a comparable scale for modeling.

### 2.5. Feature Selection Strategy

A multi-stage strategy was employed to identify a compact and generalizable set of predictors:

1. Pre-filtering: Features with near-zero variance or high multicollinearity (Pearson’s r > 0.9) were removed, reducing the set to 4381 features.
2. Univariate Evaluation: Each feature was evaluated individually using an XGBoost classifier, assessing AUC and sensitivity within each country.
3. Stratified Selection: To ensure physiological interpretability and prevent any single acoustic domain from dominating, features were categorized into families (e.g., MFCC, F0, Jitter, Shimmer). We selected the top 12 features for each country, ensuring representation across all acoustic pathways (Table 3).
4. Benchmarking with Recursive Feature Elimination: To validate our stratified feature selection method, we conducted a comparative benchmark analysis using Recursive Feature Elimination with cross-validation (RFE-CV). The performance of a model built with features selected by RFE-CV was compared to that of our primary model. This benchmark was designed to assess whether our physiologically informed selection strategy could achieve performance comparable to a standard, data-driven feature selection algorithm.
5. Cross-Cultural Identification: Features were ranked based on their minimum sensitivity across countries and the absolute difference in AUC (AUC Diff). This prioritized features that remained stable and effective in both linguistic contexts, resulting in a final set of 12 cross-cultural features.

### 2.6. Model Training and Validation

A rigorous repeated nested cross-validation framework was implemented to prevent data leakage and ensure model robustness:

1. Validation Structure: The process was repeated 30 times. Each repetition used a 5-fold outer loop for testing and a 5-fold inner loop for hyperparameter tuning. A stratified group K-fold split ensured that all utterances from a single participant remained within the same fold.
2. Class Imbalance: To address the underrepresentation of the depressed class, class weights inversely proportional to frequency were applied in the XGBoost models. The XGBoost algorithm was chosen due to its proven efficiency in handling high-dimensional data, its robustness to overfitting through regularization.
3. Metrics: Performance was evaluated using an optimized threshold (maximizing the F1-score) and averaged across all 150 test folds. DeLong’s test for AUC differences was also applied.
4. Alternative Algorithm (Robustness Check): To ensure that the performance of the identified acoustic features was not algorithm-specific and to address potential concerns about model selection, a Random Forest classifier was implemented alongside XGBoost using the identical nested cross-validation framework. This allowed for a direct comparison of the feature set’s generalizability across different modeling paradigms.
5. Interpretability: SHAP (SHapley Additive exPlanations) values were computed to quantify the contribution of each feature to the model’s predictions.

### 2.7. Statistical Control for Demographic Confounders

To assess the impact of age and sex, we performed demographic residualization. Linear regression models (feature ~ age + sex) were fitted within the control groups to calculate residuals, representing feature variance independent of demographics. Model performance using these adjusted features was compared against the original features using paired *t*-tests and Cohen’s d to verify the robustness of the biomarkers.

## 3. Results

### 3.1. Participant’s Characteristic

The demographic and clinical profiles of the participants are summarized in Table 4. The Vietnamese cohort (n = 123) exhibited a higher proportion of female participants (72.4%), whereas the Japanese cohort (n = 128) was predominantly male (53.1%). Age variance was observed between the two groups, with the Japanese cohort being notably older (53.8 ± 14.6 years) compared to the Vietnamese cohort (31.0 ± 12.0 years).

Regarding depression classification, using Cut-off 1 (No Risk vs. At Risk), 45.9% of the Vietnamese participants were identified as “At Risk,” compared to 19.5% in the Japanese cohort. Under the more stringent Cut-off 2 (Low Risk vs. High Risk), the prevalence of “High Risk” cases was 16.3% and 10.2% for the Vietnamese and Japanese datasets, respectively.

### 3.2. Feature Selection and Importance

The multi-stage feature selection process identified 12 optimal acoustic features for each country, as well as a shared set of 12 cross-culturally consistent features for Cut-off 1.

For the Vietnamese dataset, features from the Auditory Spectrum (AudSpec) and Mel-Frequency Cepstral Coefficients (MFCC) families consistently demonstrated the highest predictive power, with AUC values reaching 0.976. In contrast, the Japanese dataset was characterized by the dominance of AudSpec and Pulse Code Modulation (PCM) features, achieving AUCs up to 0.999.

The final set of 12 cross-cultural features (Table 5) exhibited high stability across both cohorts. Notably, *logHNR_sma_range* and *pcm_fftMag_psySharpness_sma_minSegLen* demonstrated high minimum sensitivity (≥0.930) and minimal performance disparity between countries (∆AUC ≤ 0.045).

Complete performance metrics for the individual country-specific and cross-cultural features are provided in Tables S3a,b and S4a,b.

SHAP analysis was employed to quantify feature contribution (Table 6). For the cross-cultural model (Cut-off 1), the three most influential features were *mfcc_sma[2]_minSegLen* (mean SHAP = 0.069), *audSpec_Rfilt_sma[17]_minSegLen* (0.042), and *pcm_fftMag_psySharpness_sma_minSegLen* (0.042). While country-specific models showed slight variations—with F0-related features ranking higher in the Vietnamese cohort—the cross-cultural features maintained substantial global importance. Detailed results of SHAP analysis for all models were provided in Supplementary Table S5a,b).

To further validate the robustness of our stratified feature selection approach, an RFE benchmark analysis was performed (Table S6). For Cut-off 1, our 12-feature set demonstrated non-inferior performance compared to both the RFE-derived 12-feature benchmark (∆AUC = +0.006) and the RFE-optimal 10-feature set (∆AUC = +0.001), while maintaining a more balanced sensitivity–specificity profile. For Cut-off 2, all methods exhibited a decline in sensitivity for identifying the ‘High Risk’ group. Although our approach achieved a higher AUC (0.944) compared to the RFE-optimal 40-feature set (AUC = 0.915), sensitivity remained constrained (0.758), reflecting the inherent challenge of classifying the limited high-severity subgroup (n = 33).

### 3.3. Model Performance Evaluation

The performance of the predictive models, evaluated via repeated nested cross-validation, is summarized in Table 7. The Cross-Cultural Model (trained on the combined dataset) demonstrated an Accuracy of 87.5%, an AUC of 0.934, and a Sensitivity of 79.9% for Cut-off 1, indicating its potential for generalizable screening applications.

When cross-cultural features were applied to individual countries, the Japanese cohort showed high classification accuracy (AUC: 0.993; Sensitivity: 91.7%), while the Vietnamese cohort retained a robust AUC of 0.913. Consistent with the optimization process, country-specific models (using features optimized for each nation) yielded the highest within-cohort performance, notably in the Japanese dataset (AUC: 0.992; Sensitivity: 93.0%).

Statistical comparison using DeLong’s test (Table S7) confirmed that the performance disparity between the two cohorts was statistically significant for both cut-offs (*p* < 0.002). This likely reflects the heterogeneous nature of the diagnostic instruments (self-report BDI vs. clinician-rated HAM-D) and varying sample characteristics.

### 3.4. Sensitivity Analysis and Robustness

To ensure the reliability of the identified biomarkers, we conducted sensitivity analyses to assess robustness against demographic confounders and variations in clinical label definition. First, adjusting the acoustic features for age and sex did not significantly alter the models’ predictive performance. The difference in AUC between the original and adjusted models was negligible (ΔAUC ≤ 0.003, *p* > 0.05), confirming that the vocal biomarkers are robust against demographic variations (Table S8). Second, the cross-cultural feature set demonstrated stability across different harmonized definitions of depression risk (Table 8). Notably, when applying the more stringent ‘Alternative Moderate’ definition (BDI ≥ 24/HAM-D ≥ 18), the model achieved an AUC of 0.980 with an exceptional specificity of 0.992, indicating enhanced discriminative power for identifying more severe and clinically unambiguous cases.

**Figure 2:**
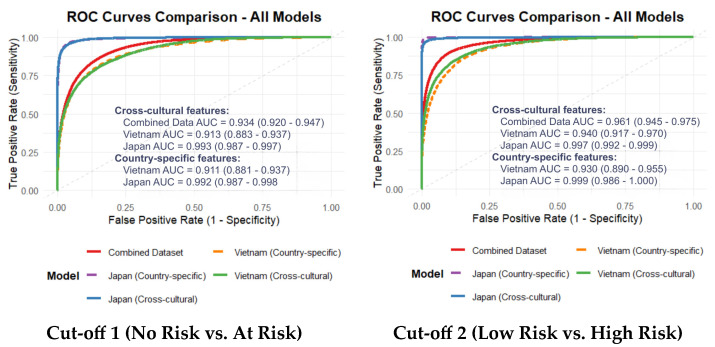
AUC of the models using cross-cultural and country-specific features.

**Figure 3:**
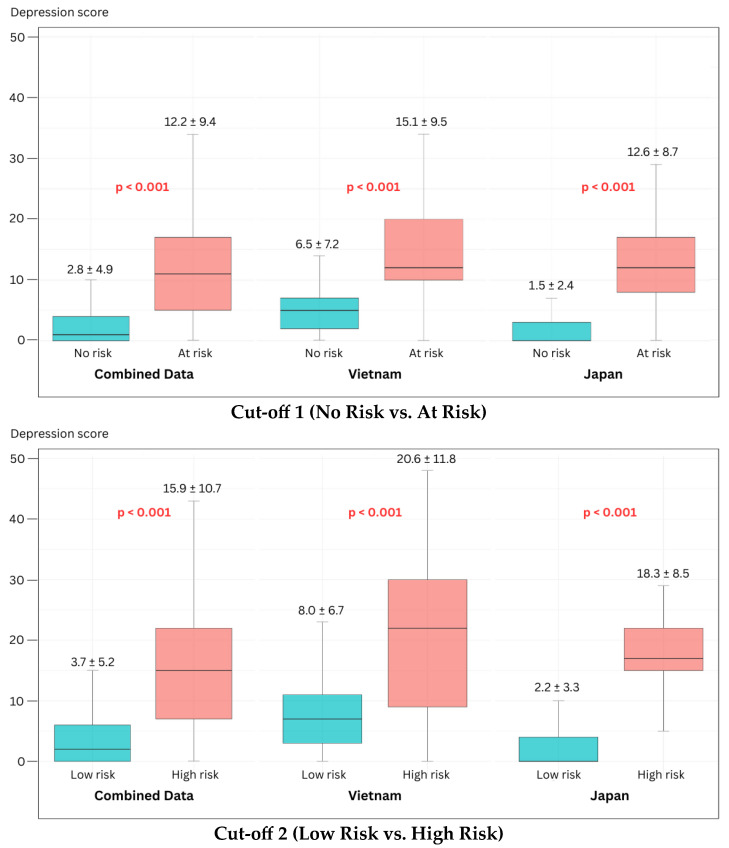
Box plots of the depression score distributions using Cross-cultural features.

As illustrated in Figure 2, the Japan-specific models exhibited the most favorable AUCs, while the cross-cultural models demonstrated consistent and robust performance across both nations. The score distributions—representing the model’s predicted depression risk—show a distinct and statistically significant separation between risk groups (Figure 3). These findings support the model’s capacity to capture severity-related acoustic patterns independent of linguistic background.

## 4. Discussion

This study addresses a critical and persistent barrier in computational psychiatry: the lack of objective, scalable, and—most importantly—cross-culturally generalizable biomarkers for depression. Our primary contribution is the identification and validation of a compact 12-feature acoustic set, predominantly comprising spectral and cepstral descriptors. This feature set demonstrated robust and consistent classification performance across Vietnamese and Japanese cohorts. While language-specific models naturally attained the highest accuracy, the derived cross-cultural feature set maintained high discriminative power (AUC > 0.90), suggesting that specific acoustic manifestations of depression transcend linguistic and phonetic boundaries.

Our analysis further revealed subtle but meaningful nuances between the two languages. Formant-related features, which are sensitive to articulatory modulation, appeared more influential in the Vietnamese cohort. This may be attributed to the complex articulatory adjustments required for Vietnamese lexical tones. Conversely, energy-related features and higher-order MFCC dynamics were more prominent in the Japanese dataset, potentially reflecting reduced vocal effort and diminished articulatory variability. These findings suggest that while the underlying pathophysiology of depression may be universal, its acoustic expression is subtly modulated by language-specific phonetic demands.

### 4.1. Language-Specific Features and the Influence of Dataset Heterogeneity

We first confirmed the feasibility of developing high-performance, language-specific models. The Vietnam-specific models achieved strong performance (AUC 0.911–0.930), while the Japanese models exhibited near-perfect classification (AUC 0.992–0.999). This performance disparity likely reflects a “label purity bias”; the Japanese dataset consisted of clinically diagnosed MDD patients versus healthy controls, whereas the Vietnamese cohort utilized a community-clinical mixed sample with self-reported BDI scores. The inherent noise and potential cultural response bias in self-reports often result in lower model performance compared to clinically gold-standard labels, a phenomenon well-documented in recent cross-lingual literature [9,29].

Across both datasets, the most discriminative features were consistently drawn from the MFCC, AudSpec, and PCM families. This corroborates extensive evidence indicating that spectral and cepstral descriptors are among the most resilient predictors of depression [10,11,30]. Specifically, the high ranking of MFCC 2 (*mfcc_sma*[2]) aligns with studies verifying its efficacy in capturing finer spectral details associated with vocal tract alterations and laryngeal tension [8]. Our results reinforce the findings of previous studies [20,21], extending the list of languages where physiologically rooted markers—such as spectral harmonicity and reduced articulation dynamics—serve as reliable indicators of psychomotor impairment.

### 4.2. Implications of the Cross-Cultural Feature Set and Robustness Analyses

The study’s core finding is the isolation of a compact set of 12 cross-cultural features that were not dominated by language-dependent prosodic elements (e.g., F0 statistics). The predominance of spectral (AudSpec) and cepstral (MFCC) features suggests that this set captures the fundamental physiological underpinnings of depression, such as neuromuscular slowing and reduced vocal tract coordination, rather than surface-level linguistic variation. SHAP analysis confirmed that features representing spectral shape and perceived sharpness (psySharpness) were the primary drivers of model decisions, likely capturing the “flat” or “muffled” vocal quality characteristic of depressive speech across cultures.

Notably, the cross-cultural model’s performance was comparable to the language-specific models within this two-country context. For the Vietnamese dataset, the cross-cultural AUC (0.913) was similar to the country-specific model (0.911), showing stable performance even at Cut-off 2. These results suggest that our feature selection approach potentially mitigates some linguistic variance by identifying features with consistent patterns across both Vietnamese and Japanese datasets. The robustness of this feature set is further supported by its consistent performance across different machine learning algorithms. While XGBoost was chosen as the primary model for its optimal balance of performance and efficiency, a comparative analysis with a Random Forest classifier revealed that the feature set maintained robust discriminative power, indicating that the predictive signal is inherent to the acoustics and not an artifact of a specific algorithm.

Supplementary analyses reinforce the validity of these findings. First, our domain-balanced feature selection approach yielded performance comparable to the data-driven RFE benchmark (AUC 0.934 vs. 0.933), while potentially offering better alignment with established biological constructs. Second, label harmonization analysis indicated that the identified acoustic signatures remained relatively stable across different diagnostic instruments (BDI vs. HAM-D), suggesting these markers may reflect clinical severity rather than scale-specific artifacts. Third, demographic residualization showed that age and sex differences did not substantially alter model performance, confirming that the selected features primarily represent variance associated with depressive symptoms.

The high AUCs (0.913–0.993) observed in our models align with a trend in the recent literature reporting exceptional classification accuracies for depression using vocal biomarkers [22,28]. However, as critically noted in systematic reviews, such high performance metrics, often derived from optimized within-dataset validation, can be misleading and frequently fail to generalize in external or cross-linguistic validation [8,9]. This underscores a fundamental challenge in computational psychiatry: distinguishing robust, generalizable biomarkers from statistical patterns that overfit to specific datasets.

The performance disparity between our Japanese (near-perfect AUC) and Vietnamese (high but lower AUC) cohorts illustrates this challenge. While superior “label purity” from clinician-rated diagnoses in the Japanese sample is a primary explanation, alternative interpretations related to cultural and methodological biases must be considered. The self-report nature of the BDI in the Vietnamese cohort introduces potential cultural response biases, where individuals may under-report or over-report symptoms due to stigma or differing cultural expressions of distress, thereby increasing “label noise” and attenuating model performance [9,23]. This heterogeneity in ground truth definition itself represents a key challenge for the field, complicating direct comparisons.

Furthermore, the risk of overfitting extends beyond labels to linguistic and recording artifacts. The field has documented instances where features seemingly robust in one linguistic context fail in another, as seen in the language-specificity of pausing behaviors in the RADAR-MDD study [20] or the significant performance drop of self-supervised learning features in cross-lingual tasks [29]. A truly balanced view acknowledges that high AUCs, while encouraging, are not definitive proof of a biomarker’s validity. They must be tempered by the recognition that our validation, while cross-cultural, remains limited to two languages and specific dataset characteristics. Therefore, while this study identifies a promising, physiologically informed feature set, its performance must be interpreted as a strong initial signal within a defined context, rather than as a fully validated universal solution. This finding aligns with the objectives of advanced methodologies like domain adaptation and multi-lingual feature selection [21,31], but definitive establishment of these features as language-invariant requires further validation across a broader range of languages and controlled settings.

### 4.3. Strengths and Limitations

A major strength of this study is the validation of a non-inferior, cross-cultural feature set that facilitates scalable, low-cost screening in resource-constrained settings like Vietnam. A vocal biomarker that does not require extensive language-specific recalibration could significantly accelerate the deployment of mobile health interventions in diverse communities.

However, several limitations warrant consideration. First, the “ground-truth mismatch” between self-report and clinician-rated instruments remains a confounding factor in direct severity comparisons. Second, while standardized, the different recording protocols and number of phrases between datasets may introduce acoustic variability. Third, the use of scripted speech may not capture the full range of spontaneous affective expression. Fourth, the small sample size of the “High Risk” group (n = 33) limits the reliability of high-severity classification, suggesting that sensitivity metrics for severe cases should be interpreted with caution until validated in larger, prospective cohorts. This is evidenced by our RFE benchmark analysis, where both physiologically informed and data-driven approaches showed suboptimal sensitivity (0.628–0.758) for Cut-off 2. These results suggest that while our core features are cross-culturally stable for general screening, further refinement with larger clinical samples is essential to improve the detection of severe depression. A further methodological consideration for future research is the formal design of falsifiability tests—such as evaluating model performance on cohorts with comorbid non-psychiatric voice disorders or under acoustically adversarial conditions—to rigorously challenge the specificity and robustness of the proposed acoustic biomarkers beyond the current validation framework.

### 4.4. Future Directions

Building on these findings, several critical research avenues are proposed to validate and translate the identified biomarkers. First, prospective multilingual trials are essential, incorporating a third, typologically distinct language family (e.g., Indo-European) with fully standardized recording and clinical assessment protocols to rigorously test the universality of the 12-feature set. Second, longitudinal studies are needed to determine if these features act as state-dependent markers sensitive to treatment response, thereby enhancing their utility for monitoring clinical trajectories. Third, technical robustness must be advanced through the implementation of domain adaptation techniques (e.g., adversarial training, self-supervised learning) to minimize performance shifts caused by linguistic and environmental variability. Crucially, future work must also incorporate falsifiability tests—such as evaluating model performance on cohorts with non-psychiatric voice disorders (e.g., laryngitis, dysarthria) or under acoustically adversarial conditions—to challenge the specificity of the features to depression and safeguard against overfitting. Finally, research should progress toward real-world integration, conducting feasibility and acceptability studies for deploying voice-based screening in primary care or telehealth settings. This integrated pathway, from rigorous validation to practical implementation, aims to advance the development of clinically reliable, generalizable, and equitable digital mental health tools.

## 5. Conclusions

In conclusion, this study identifies a compact set of acoustic vocal biomarkers—predominantly spectral and cepstral parameters—that demonstrate robust discriminative capacity for depression across linguistically and culturally distinct cohorts. The successful classification of depressive states in both Vietnamese and Japanese participants using a unified 12-feature set provides empirical support for a core “physiological voice signature” of depression, potentially transcending language-specific phonetic variations. While the cross-cultural model achieved high performance (AUC > 0.90), these results must be contextualized within key methodological heterogeneities, including the reliance on different diagnostic instruments (self-report BDI vs. clinician-rated HAM-D) and varying recording protocols, which may introduce cultural response biases and acoustic confounders. Nevertheless, the maintained predictive integrity of specific acoustic markers across these disparities highlights the potential resilience of vocal biomarkers. These findings hold promise for global mental health equity, particularly in low-resource settings, by suggesting a pathway toward non-invasive, cost-effective screening tools that may require less language-specific recalibration.

## Figures and Tables

**Table 1:** Classification of depression risk among participants.

Severity	BDI Score	HAM-D Score	Cut-Off 1	Cut-Off 2
Normal/Minimal	0–9	0–7	No risk	Low risk
Mild Depression	10–18	8–13	At risk	Low risk
Moderate Depression	19–29	14–18	At risk	High risk
Severe/Very Severe	≥30	≥19	At risk	High risk

**Table 2:** Voice recording characteristics of the datasets.

Characteristic	Vietnam Dataset	Japan Dataset
Number of Unique Phrases	16	21
Total Number of Utterances	2091	4910
Utterance Repetition	No	Yes

**Table 3:** Distribution of pre-filtered feature families and the final selection of 12 acoustic features.

No	Feature Family	Frequency	Percentage	Number of Features Selected
1	Audspec	1756	40%	3
2	MFCC	1143	26%	2
3	PCM	1135	26%	2
4	Jitter	112	3%	1
5	F0	67	2%	1
6	logHNR	53	1%	1
7	Shimmer	57	1%	1
8	Voicing	58	1%	1
	**Total**	**4381**	**100%**	**12**

**Table 4:** Participant’s characteristics.

Characteristics	Vietnam (n = 123)	Japan (n = 128)	Combined Data (n = 251)
Age, mean (sd)	31.0 (12.0)	53.8 (14.6)	42.6 (17.5)
Sex, n (%)			
Male	34 (27.6)	68 (53.1)	102 (40.6)
Female	89 (72.4)	60 (46.9)	149 (59.4)
Depression classification—Cutoff 1, n (%)			
No risk	69 (56.1)	103 (80.5)	172 (68.5)
At risk	54 (45.9)	25 (19.5)	79 (31.5)
Depression classification—Cutoff 2, n (%)			
Low risk	103 (83.7)	115 (89.8)	218 (86.9)
High risk	20 (16.3)	13 (10.2)	33 (13.1)

**Table 5:** Cross-cultural acoustic features selected using Cut-off 1.

No.	Feature Family	Feature Selected	Min. Sen.	∆AUC
1	Audspec	audSpec_Rfilt_sma[17]_minSegLen	0.954	0.045
2		audSpec_Rfilt_sma[15]_minSegLen	0.926	0.037
3		audSpec_Rfilt_sma[20]_minSegLen	0.920	0.035
4	F0	F0final_sma_qregerrQ	0.700	0.012
5	Jitter	jitterDDP_sma_de_lpc4	0.722	0.009
6	logHNR	logHNR_sma_range	0.845	0.045
7	MFCC	mfcc_sma[2]_minSegLen	0.936	0.067
8		mfcc_sma[1]_minSegLen	0.883	0.025
9	PCM	pcm_fftMag_psySharpness_sma_minSegLen	0.932	0.028
10		pcm_fftMag_spectralHarmonicity_sma_minSegLen	0.930	0.027
11	Shimmer	shimmerLocal_sma_risetime	0.704	0.013
12	Voicing	voicingFinalUnclipped_sma_lpc0	0.799	0.061

Min. Sen.: Minimum sensitivity; ∆AUC: Difference in AUC; Audspec: auditory-spectrum; F0: Fundamental Frequency; logHNR: log Harmonics-to-Noise Ratio; MFCC: (Mel-Frequency Cepstral Coefficients; PCM: Pulse Code Modulation.

**Table 6:** SHAP-based feature importance rankings of cross-cultural acoustic features (Cut-off 1).

No.	Feature Name	SHAP, Mean (sd)
1	mfcc_sma[2]_minSegLen	0.069 (0.009)
2	audSpec_Rfilt_sma[17]_minSegLen	0.042 (0.004)
3	pcm_fftMag_psySharpness_sma_minSegLen	0.042 (0.008)
4	mfcc_sma[1]_minSegLen	0.039 (0.005)
5	pcm_fftMag_spectralHarmonicity_sma_minSegLen	0.036 (0.005)
6	logHNR_sma_range	0.035 (0.004)
7	audSpec_Rfilt_sma[20]_minSegLen	0.027 (0.003)
8	audSpec_Rfilt_sma[15]_minSegLen	0.023 (0.004)
9	voicingFinalUnclipped_sma_lpc0	0.006 (0.003)
10	F0final_sma_qregerrQ	0.005 (0.001)
11	jitterDDP_sma_de_lpc4	0.001 (0.001)
12	shimmerLocal_sma_risetime	0.001 (0.000)

**Table 7:** Model performance summary.

Model Type	AUC	Accu.	F1	Sens.	Spec.	TP	TN	FP	FN
**Cut-off 1 (No Risk vs. At Risk)**									
***XGBoost***									
**Cross-Cultural Features**									
Combined Data	0.934	0.875	0.772	0.799	0.903	296	930	100	74
Vietnam	0.913	0.833	0.816	0.843	0.825	155	194	41	29
Japan	0.993	0.973	0.926	0.917	0.985	171	784	12	15
**Country-Specific Features**									
Vietnam	0.911	0.837	0.820	0.850	0.826	156	194	41	27
Japan	0.992	0.973	0.929	0.930	0.983	173	783	13	13
***RandomForest***									
**Cross-Cultural Features**									
Combined Data	0.898	0.853	0.728	0.743	0.892	297	981	73	50
Vietnam	0.791	0.701	0.704	0.810	0.613	155	187	28	48
Japan	0.952	0.932	0.815	0.775	0.970	166	787	21	9
**Country-Specific Features**									
Vietnam	0.742	0.650	0.674	0.830	0.507	153	194	31	41
Japan	0.976	0.959	0.888	0.850	0.984	179	793	8	3
**Cut-off 2 (Low Risk vs. High Risk)**									
***XGBoost***									
**Cross-Cultural Features**									
Combined Data	0.944	0.961	0.759	0.758	0.969	124	1199	39	39
Vietnam	0.918	0.940	0.745	0.741	0.952	51	333	17	17
Japan	0.992	0.997	0.958	0.948	0.997	90	884	3	5
**Country-Specific Features**									
Vietnam	0.900	0.930	0.707	0.737	0.932	50	326	24	18
Japan	0.996	0.999	0.981	0.977	0.998	93	886	2	2
***RandomForest***									
**Cross-Cultural Features**									
Combined Data	0.889	0.921	0.648	0.618	0.961	294	953	76	78
Vietnam	0.811	0.834	0.538	0.589	0.880	152	184	31	50
Japan	0.954	0.964	0.812	0.787	0.983	166	784	20	12
**Country-Specific Features**									
Vietnam	0.777	0.833	0.503	0.522	0.891	154	156	30	78
Japan	0.981	0.996	0.979	0.972	0.999	175	791	11	5

AUC: Area Under the Curve; Accu.: accuracy; F1: F1 score; Sens.: sensitivity; Spec.: specificity; TP: true positive; TN: true negative; FP: false positive; FN: false negative.

**Table 8:** Classification performance of different depression label definitions (cross-cultural features).

Label Definition	AUC	Accu.	F1	Sens.	Spec.
Alternative Mild (BDI ≥ 14/HAM-D ≥ 11)	0.958	0.925	0.767	0.762	0.957
Original Mild (BDI ≥ 10/HAM-D ≥ 8)	0.934	0.875	0.772	0.799	0.903
Alternative Moderate (BDI ≥ 24/HAM-D ≥ 18)	0.980	0.977	0.823	0.771	0.992
Original Moderate (BDI ≥ 19/HAM-D ≥ 14)	0.961	0.944	0.759	0.758	0.969

AUC: Area Under the Curve; Accu.: accuracy; F1: F1 score; Sens.: sensitivity; Spec.: specificity.

## Data Availability

The data presented in this study are available on request from the corresponding author. The data are not publicly available due to privacy and ethical restrictions, as the dataset contains voice recordings, which are biometric identifiers. Access to the anonymized data is restricted to ensure participant confidentiality.

## Supplementary Materials

The following supporting information can be downloaded at https://www.mdpi.com/article/10.3390/bioengineering13010033/s1. Table S1. Post hoc power analysis for ROC-based classification; Table S2. Set of pre-defined phrases used in voice recording; Table S3a. Cross-cultural acoustic features selected using Cut-off 1; Table S3b. Country-specific acoustic features selected for Cut-off 1; Table S4a. Country-specific acoustic features selected for Cut-off 2; Table S4b. Cross-cultural acoustic features selected for Cut-off 2; Table S5a. SHAP-based feature importance rankings (Cut-off 1); Table S5b. SHAP-based feature importance rankings (Cut-off 2); Table S6. RFE validation of feature selection; Table S7. Statistical comparison of AUC values using DeLong’s test; Table S8. Sensitivity analysis for age and sex confounders.

## Abbreviations

The following abbreviations are used in this manuscript:

AUC	Area Under the Curve
AudSpec	Auditory Spectrum
BDI	Beck Depression Inventory
F0	Fundamental Frequency
HAM-D	Hamilton Depression Rating Scale
LMICs	Low- and Middle-Income Countries
logHNR	Logarithmic Harmonics-to-Noise Ratio
MDD	Major Depressive Disorder
MFCCs	Mel-Frequency Cepstral Coefficients
ml-MRMR	multi-lingual Minimum Redundancy Maximum Relevance
PCM	Pulse Code Modulation
RFE-CV	Recursive Feature Elimination with Cross-Validation
SHAP	SHapley Additive exPlanations
