# Supplementary material for: Cross-Cultural Identification of Acoustic Voice Features for Depression: A Cross-Sectional Study of Vietnamese and Japanese Datasets
Source: Bioengineering (Basel). 2025 Dec 27;13(1):33. doi: 10.3390/bioengineering13010033 (PMC12837578; doi:10.3390/bioengineering13010033)
Supplement: Supplementary file 1 [file bioengineering-13-00033-s001.zip › bioengineering-4042676-supplementary.pdf]

Supplementary Materials

Table S1. Post hoc power analysis for ROC-based classification

Model	Dataset	n_cases	n_controls	Observed AUC	Power
Cut-off 1					
**Cross-Cultural Features**					
XGBoost	Combined	1849	5152	0.933	1.00
XGBoost	Vietnam	918	1173	0.910	1.00
XGBoost	Japan	931	3979	0.992	1.00
**Country-Specific Features**					
XGBoost	Vietnam (Spec)	918	1173	0.909	1.00
XGBoost	Japan (Spec)	931	3979	0.993	1.00
Cut-off 2					
**Cross-Cultural Features**					
XGBoost	Combined	814	6187	0.960	1.00
XGBoost	Vietnam	340	1751	0.944	1.00
XGBoost	Japan	474	4436	0.996	1.00
**Country-Specific Features**					
XGBoost	Vietnam (Spec)	340	1751	0.923	1.00
XGBoost	Japan (Spec)	474	4436	0.998	1.00

Table S2. Set of pre-defined phrases used in voice recording

Japanese	Vietnamese	English translation
いろはにほへと<br>(i ro ha ni ho he to)	alpha beta gamma delta	
本日は晴天なり<br>(honjitsu wa seitennari)	Hôm nay là một ngày nắng	It is sunny today
つれづれなるままに<br>(tsurezure naru mama ni)		Just as it is
吾輩は猫である<br>(wagahai wa nekodearu)		I am a cat
むかしむかしあるところに<br>(mukashi mukashi aru tokoro ni)	Ngày xửa ngày xưa	Once upon a time
あいうえお<br>(a i u e o)	A B C D E F G	A B C D E F G
らりるれろ		Ra ri ru re ro

Japanese	Vietnamese	English translation
(ra ri ru re ro)		
ぱぴぷぺぽ<br>(pa pi pu pe po)		Pa pi pu pe po
かきくけこ<br>(ka ki ku ke ko)		Ka ki ku ke ko
思えば遠くへ来たもんだ<br>(omoeba tōku e kita monda)		Now that I think about it, I've come a long way
ガラパゴス諸島<br>(garapagosu shotō)	Quần đảo Ga-la-pa-gốt	Galapagos Islands
疲れてぐったりしています<br>(tsukarete guttari shiteimasu)	Tôi cảm thấy mệt mỏi và kiệt sức	I'm tired and exhausted
とても元気です<br>(totemo genkidesu)	Tôi đang rất ổn	I'm very well
昨日はよく眠れました<br>(kinō wa yoku nemuremashit a)	Hôm qua tôi có một giấc ngủ ngon	I slept well yesterday
食欲があります<br>(shokuyoku ga arimasu)	Tôi có cảm giác thèm ăn	I have an appetite
怒りっぽいです<br>(okorippoi desu)	Tôi cảm thấy cáu kỉnh	I'm angry
心が穏やかです<br>(kokoro ga odayaka desu)	Tôi cảm thấy tĩnh tâm	My heart is calm
	Tôi luôn nhìn về phía trước	I always looked ahead
	Cố lên nào tôi ơi	Never, never give up
あー (a –)	a –	[aː]
えー (e –)	e –	[æː]
うー (u –)	u –	[uː]
ぱたかぱたか・・<br>(pa ta ka pa ta ka)	pa-ta-ka, pa-ta-ka,…	pa-ta-ka, pa-ta-ka,…

Table S3a. Cross-cultural acoustic features selected using Cut-off 1

No.	Feature family	Feature selected	Min. Sen.	ΔAUC
1	Audspec	audSpec_Rfilt_sma[17]_minSegLen	0.954	0.045
2		audSpec_Rfilt_sma[15]_minSegLen	0.926	0.037
3		audSpec_Rfilt_sma[20]_minSegLen	0.920	0.035
4	F0	F0final_sma_qregerrQ	0.700	0.012

No.	Feature family	Feature selected	Min. Sen.	ΔAUC
5	Jitter	jitterDDP_sma_de_lpc4	0.722	0.009
6	logHNR	logHNR_sma_range	0.845	0.045
7	MFCC	mfcc_sma[2]_minSegLen	0.936	0.067
8		mfcc_sma[1]_minSegLen	0.883	0.025
9	PCM	pcm_fftMag_psySharpness_sma_minSegLen	0.932	0.028
10		pcm_fftMag_spectralHarmonicity_sma_minSegLen	0.930	0.027
11	Shimmer	shimmerLocal_sma_risetime	0.704	0.013
12	Voicing	voicingFinalUnclipped_sma_lpc0	0.799	0.061

Min. Sen.: Minimum sensitivity; ΔAUC: Difference in AUC; Audspec: auditory-spectrum; F0: Fundamental Frequency; logHNR: log Harmonics-to-Noise Ratio; MFCC: (Mel-Frequency Cepstral Coefficients; PCM: Pulse Code Modulation.

Table S3b. Country-specific acoustic features selected for Cut-off 1

No.	Feature family	Feature selected	Sensitivity	AUC
		**Vietnam**		
1	Audspec	audSpec_Rfilt_sma[17]_minSegLen	0.954	0.953
2		audSpec_Rfilt_sma[18]_minSegLen	0.927	0.941
3		audSpec_Rfilt_sma[15]_minSegLen	0.926	0.957
4	F0	F0final_sma_ff0_minSegLen	0.891	0.921
5	Jitter	jitterLocal_sma_iqr2-3	0.897	0.812
6	logHNR	logHNR_sma_range	0.903	0.917
7	MFCC	mfcc_sma_de[1]_peakMeanRel	0.974	0.970
8		mfcc_sma_de[2]_peakMeanRel	0.950	0.976
9	PCM	pcm_zcr_sma_de_minSegLen	0.937	0.964
10		pcm_fftMag_psySharpness_sma_minSegLen	0.932	0.969
11	Shimmer	shimmerLocal_sma_maxPos	0.881	0.837
12	Voicing	voicingFinalUnclipped_sma_flatness	0.890	0.926
		**Japanese**		
1	Audspec	audSpec_Rfilt_sma[23]_minSegLen	0.983	0.993
2		audSpec_Rfilt_sma_de[22]_minSegLen	0.980	0.998
3		audSpec_Rfilt_sma_de[5]_minSegLen	0.977	0.999
4	F0	F0final_sma_iqr2-3	0.725	0.807
5	Jitter	jitterDDP_sma_de_amean	0.722	0.804
6	logHNR	logHNR_sma_range	0.845	0.961
7	MFCC	mfcc_sma[14]_minSegLen	0.971	0.992
8		mfcc_sma[2]_minSegLen	0.939	0.996

No.	Feature family	Feature selected	Sensitivity	AUC
9	PCM	pcm_fftMag_fband1000-4000_sma_de_minSegLen	0.984	0.996
10		pcm_fftMag_psySharpness_sma_de_minSegLen	0.977	0.995
11	Shimmer	shimmerLocal_sma_risetime	0.704	0.842
12	Voicing	voicingFinalUnclipped_sma_lpc0	0.799	0.965

Min. Sen.: Minimum sensitivity; ΔAUC: Difference in AUC; Audspec: auditory-spectrum; F0: Fundamental Frequency; logHNR: log Harmonics-to-Noise Ratio; MFCC: (Mel-Frequency Cepstral Coefficients; PCM: Pulse Code Modulation.

Table S4a. Country-specific acoustic features selected for Cut-off 2

No.	Feature family	Feature selected	Sensitivity	AUC
		**Vietnam**		
1	Audspec	audSpec_Rfilt_sma[8]_minSegLen	0.912	0.975
2		audSpec_Rfilt_sma[1]_minSegLen	0.900	0.975
3		audSpec_Rfilt_sma[24]_minSegLen	0.894	0.981
4	F0	F0final_sma_ff0_minSegLen	0.776	0.951
5	Jitter	jitterDDP_sma_linregc1	0.765	0.857
6	logHNR	logHNR_sma_lpc4	0.759	0.855
7	MFCC	mfcc_sma_de[1]_peakMeanRel	0.935	0.987
8		mfcc_sma_de[2]_peakMeanRel	0.894	0.987
9	PCM	pcm_fftMag_spectralHarmonicity_sma_minSegLen	0.918	0.979
10		pcm_fftMag_fband250-650_sma_minSegLen	0.906	0.983
11	Shimmer	shimmerLocal_sma_de_lpc4	0.791	0.865
12	Voicing	voicingFinalUnclipped_sma_flatness	0.794	0.950
		**Japanese**		
1	Audspec	audSpec_Rfilt_sma_de[18]_minSegLen	0.992	0.998
2		audSpec_Rfilt_sma_de[20]_minSegLen	0.992	0.994
3		audSpec_Rfilt_sma[8]_minSegLen	0.989	0.999
4	F0	F0final_sma_de_upleveltime25	0.711	0.883
5	Jitter	jitterDDP_sma_lpc0	0.629	0.853
6	logHNR	logHNR_sma_pctlrange0-1	0.865	0.980
7	MFCC	mfcc_sma[12]_minSegLen	0.985	0.994
8		mfcc_sma[14]_minSegLen	0.975	0.995
9	PCM	pcm_fftMag_spectralRollOff75.0_sma_de_minSegLen	0.994	0.996
10		pcm_fftMag_fband250-650_sma_de_peakMeanRel	0.989	0.998
11	Shimmer	shimmerLocal_sma_de_upleveltime90	0.622	0.851
12	Voicing	voicingFinalUnclipped_sma_flatness	0.806	0.980

Min. Sen.: Minimum sensitivity; ΔAUC: Difference in AUC; Audspec: auditory-spectrum; F0: Fundamental Frequency; logHNR: log Harmonics-to-Noise Ratio; MFCC: (Mel-Frequency Cepstral Coefficients; PCM: Pulse Code Modulation.

Table S4b. Cross-cutural acoustic features selected for Cut-off 2

No.	Feature family	Feature selected	Sensitivity	AUC
1	Audspec	audSpec_Rfilt_sma[8]_minSegLen	0.918	0.024
2		audSpec_Rfilt_sma[1]_minSegLen	0.900	0.020
3		audSpec_Rfilt_sma[24]_minSegLen	0.894	0.018
4	F0	F0final_sma_de_upleveltime25	0.703	0.013
5	Jitter	jitterDDP_sma_de_upleveltime75	0.616	0.042
6	logHNR	logHNR_sma_pctlrange0-1	0.753	0.024
7	MFCC	mfcc_sma[1]_minSegLen	0.856	0.016
8		mfcc_sma[12]_minSegLen	0.841	0.082
9	PCM	pcm_fftMag_spectralHarmonicity_sma_minSegLen	0.918	0.0204
10		pcm_fftMag_fband250-650_sma_minSegLen	0.906	0.016
11	Shimmer	shimmerLocal_sma_de_upleveltime75	0.616	0.007
12	Voicing	voicingFinalUnclipped_sma_flatness	0.794	0.031

Min. Sen.: Minimum sensitivity; ΔAUC: Difference in AUC; Audspec: auditory-spectrum; F0: Fundamental Frequency; logHNR: log Harmonics-to-Noise Ratio; MFCC: (Mel-Frequency Cepstral Coefficients; PCM: Pulse Code Modulation.

Table S5a. SHAP-based feature importance rankings (Cut-off 1)

No.	Feature name	SHAP, mean (sd)
**Cross-cultural features**		
1	mfcc_sma[2]_minSegLen	0.069 (0.009)
2	audSpec_Rfilt_sma[17]_minSegLen	0.042 (0.004)
3	pcm_fftMag_psySharpness_sma_minSegLen	0.042 (0.008)
4	mfcc_sma[1]_minSegLen	0.039 (0.005)
5	pcm_fftMag_spectralHarmonicity_sma_minSegLen	0.036 (0.005)
6	logHNR_sma_range	0.035 (0.004)
7	audSpec_Rfilt_sma[20]_minSegLen	0.027 (0.003)
8	audSpec_Rfilt_sma[15]_minSegLen	0.023 (0.004)
9	voicingFinalUnclipped_sma_lpc0	0.006 (0.003)
10	F0final_sma_qregerrQ	0.005 (0.001)
11	jitterDDP_sma_de_lpc4	0.001 (0.001)

No.	Feature name	SHAP, mean (sd)
**Cross-cultural features**		
12	shimmerLocal_sma_risetime	0.001 (0.000)
**Vietnam-specific features**		
1	F0final_sma_ff0_minSegLen	0.055 (0.009)
2	audSpec_Rfilt_sma[15]_minSegLen	0.049 (0.007)
3	mfcc_sma_de[1]_peakMeanRel	0.048 (0.008)
4	audSpec_Rfilt_sma[17]_minSegLen	0.046 (0.007)
5	mfcc_sma_de[2]_peakMeanRel	0.037 (0.005)
6	pcm_fftMag_psySharpness_sma_minSegLen	0.037 (0.007)
7	pcm_zcr_sma_de_minSegLen	0.031 (0.002)
8	shimmerLocal_sma_maxPos	0.019 (0.003)
9	voicingFinalUnclipped_sma_flatness	0.019 (0.003)
10	audSpec_Rfilt_sma[18]_minSegLen	0.017 (0.005)
11	jitterLocal_sma_iqr2-3	0.009 (0.005)
12	logHNR_sma_range	0.006 (0.003)
**Japan-specific features**		
1	mfcc_sma[2]_minSegLen	0.076 (0.004)
2	audSpec_Rfilt_sma_de[22]_minSegLen	0.057 (0.004)
3	audSpec_Rfilt_sma[23]_minSegLen	0.053 (0.005)
4	pcm_fftMag_psySharpness_sma_de_minSegLen	0.053 (0.005)
5	mfcc_sma[14]_minSegLen	0.050 (0.004)
6	audSpec_Rfilt_sma_de[5]_minSegLen	0.043 (0.007)
7	pcm_fftMag_fband1000-4000_sma_de_minSegLen	0.027 (0.004)
8	logHNR_sma_range	0.025 (0.003)
9	F0final_sma_iqr2-3	0.005 (0.002)
10	voicingFinalUnclipped_sma_lpc0	0.001 (0.001)
11	jitterDDP_sma_de_amean	0.000 (0.001)
12	shimmerLocal_sma_risetime	0.000 (0.000)

F0: Fundamental Frequency; logHNR: log Harmonics-to-Noise Ratio; MFCC: (Mel-Frequency Cepstral Coefficients; PCM: Pulse Code Modulation.

Table S5b. SHAP-based feature importance rankings (Cut-off 2)

No.	Feature name	SHAP, mean (sd)
**Cross-cultural features**		
1	mfcc_sma[12]_minSegLen	0.067 (0.007)
2	shimmerLocal_sma_de_upleveltime75	0.044 (0.007)

No.	Feature name	SHAP, mean (sd)
**Cross-cultural features**		
3	audSpec_Rfilt_sma[8]_minSegLen	0.043 (0.004)
4	mfcc_sma[1]_minSegLen	0.042 (0.006)
5	audSpec_Rfilt_sma[1]_minSegLen	0.04 (0.004)
6	F0final_sma_de_upleveltime25	0.038 (0.006)
7	pcm_fftMag_spectralHarmonicity_sma_minSegLen	0.032 (0.004)
8	pcm_fftMag_fband250-650_sma_minSegLen	0.019 (0.005)
9	logHNR_sma_pctlrange0-1	0.018 (0.003)
10	audSpec_Rfilt_sma[24]_minSegLen	0.016 (0.005)
11	jitterDDP_sma_de_upleveltime75	0.008 (0.003)
12	voicingFinalUnclipped_sma_flatness	0.005 (0.002)
**Vietnam-specific features**		
1	pcm_fftMag_fband250-650_sma_minSegLen	0.057 (0.005)
2	F0final_sma_ff0_minSegLen	0.047 (0.007)
3	audSpec_Rfilt_sma[24]_minSegLen	0.042 (0.008)
4	mfcc_sma_de[1]_peakMeanRel	0.037 (0.006)
5	mfcc_sma_de[2]_peakMeanRel	0.037 (0.007)
6	audSpec_Rfilt_sma[8]_minSegLen	0.032 (0.004)
7	pcm_fftMag_spectralHarmonicity_sma_minSegLen	0.032 (0.005)
8	audSpec_Rfilt_sma[1]_minSegLen	0.03 (0.007)
9	voicingFinalUnclipped_sma_flatness	0.014 (0.005)
10	shimmerLocal_sma_de_lpc4	0.014 (0.003)
11	jitterDDP_sma_linregc1	0.009 (0.004)
12	logHNR_sma_lpc4	0.008 (0.003)
**Japan-specific features**		
1	audSpec_Rfilt_sma_de[18]_minSegLen	0.047 (0.006)
2	mfcc_sma[14]_minSegLen	0.043 (0.006)
3	pcm_fftMag_spectralRollOff75.0_sma_de_minSegLen	0.043 (0.005)
4	mfcc_sma[12]_minSegLen	0.041 (0.006)
5	F0final_sma_de_upleveltime25	0.024 (0.003)
6	audSpec_Rfilt_sma[8]_minSegLen	0.019 (0.004)
7	audSpec_Rfilt_sma_de[20]_minSegLen	0.019 (0.003)
8	pcm_fftMag_fband250-650_sma_de_peakMeanRel	0.012 (0.003)
9	shimmerLocal_sma_de_upleveltime90	0.009 (0.002)
10	logHNR_sma_pctlrange0-1	0.005 (0.002)
11	voicingFinalUnclipped_sma_flatness	0.002 (0.001)
12	jitterDDP_sma_lpc0	0.001 (0.001)

F0: Fundamental Frequency; logHNR: log Harmonics-to-Noise Ratio; MFCC: (Mel-Frequency Cepstral Coefficients; PCM: Pulse Code Modulation.

Table S6. RFE validation of feature selection

Method (no. of features)	AUC	Accuracy	F1	Sensitivity	Specificity
**Cut-off 1**					
This study (12)	0.934	0.875	0.772	0.799	0.903
RFE Benchmark (12)	0.928	0.840	0.741	0.870	0.828
RFE Optimal (10)	0.933	0.856	0.755	0.840	0.862
**Cut-off 2**					
This study (12)	0.944	0.961	0.759	0.758	0.969
RFE Benchmark (12)	0.862	0.845	0.485	0.628	0.874
RFE Optimal (40)	0.915	0.895	0.591	0.653	0.927

Table S7. Statistical comparison of AUC values using DeLong's test

Comparison	Model	Cut-off	AUC (VN)	AUC (JP)	ΔAUC (JP−VN)	p-value
VN vs JP	XGBoost	Cut-off 1	0.904	0.994	+0.090	< 0.001
VN vs JP	XGBoost	Cut-off 2	0.967	0.998	+0.030	0.0015
VN vs JP	Random Forest	Cut-off 1	0.755	0.955	+0.200	< 0.001
VN vs JP	Random Forest	Cut-off 2	0.824	0.939	+0.116	< 0.001

Table S8. Sensitivity analysis for age and sex confounders

Data	Model	AUC, Mean (SD)	Difference	P - value	Cohen's d
**Cut-off 1**					
**Cross-cultural features**					
Combined	Original	0.934 (0.008)	-		
	Adjusted	0.936 (0.009)	0.002 (0.003)	0.043	0.575
Vietnam	Original	0.913 (0.015)	-		
	Adjusted	0.914 (0.016)	0.001 (0.006)	0.655	0.118
Japan	Original	0.993 (0.003)	-		
	Adjusted	0.992 (0.005)	-0.001 (0.003)	0.327	-0.262
**Country-specific features**					
Vietnam	Original	0.911 (0.017)	-		

	Adjusted	0.907 (0.022)	0.003 (0.006)	0.274	0.294
Japan	Original	0.992 (0.004)	-		
	Adjusted	0.991 (0.004)	0.000 (0.000)	0.473	0.103
**Cut-off 2**					
**Cross-cultural features**					
Combined	Original	0.934 (0.008)	-		
	Adjusted	0.936 (0.009)	0.002 (0.003)	0.043	0.575
Vietnam	Original	0.913 (0.015)	-		
	Adjusted	0.914 (0.016)	0.001 (0.006)	0.655	0.118
Japan	Original	0.993 (0.003)	-		
	Adjusted	0.992 (0.005)	-0.001 (0.003)	0.327	-0.262
**Country-specific features**					
Vietnam	Original	0.911 (0.017)	-		
	Adjusted	0.907 (0.022)	0.003 (0.006)	0.274	0.294
Japan	Original	0.992 (0.004)	-		
	Adjusted	0.991 (0.004)	0.000 (0.000)	0.473	0.103
